# Human Coronavirus HKU1 Infection of Primary Human Type II Alveolar Epithelial Cells: Cytopathic Effects and Innate Immune Response

**DOI:** 10.1371/journal.pone.0070129

**Published:** 2013-07-24

**Authors:** Samuel R. Dominguez, Emily A. Travanty, Zhaohui Qian, Robert J. Mason

**Affiliations:** 1 Department of Pediatrics, University of Colorado School of Medicine, Anschutz Medical Campus, Aurora, Colorado, United States of America; 2 Department of Microbiology, University of Colorado School of Medicine, Anschutz Medical Campus, Aurora, Colorado, United States of America; 3 Department of Medicine, National Jewish Health, Denver, Colorado, United States of America; The University of Hong Kong, China

## Abstract

Because they are the natural target for respiratory pathogens, primary human respiratory epithelial cells provide the ideal *in vitro* system for isolation and study of human respiratory viruses, which display a high degree of cell, tissue, and host specificity. Human coronavirus HKU1, first discovered in 2005, has a worldwide prevalence and is associated with both upper and lower respiratory tract disease in both children and adults. Research on HCoV-HKU1 has been difficult because of its inability to be cultured on continuous cell lines and only recently it was isolated from clinical specimens using primary human, ciliated airway epithelial cells. Here we demonstrate that HCoV-HKU1 can infect and be serially propagated in primary human alveolar type II cells at the air-liquid interface. We were not able to infect alveolar type I-like cells or alveolar macrophages. Type II alveolar cells infected with HCoV-HKU1 demonstrated formation of large syncytium. At 72 hours post inoculation, HCoV-HKU1 infection of type II cells induced increased levels of mRNAs encoding IL29,CXCL10, CCL5, and IL-6 with no significant increases in the levels of IFNβ. These studies demonstrate that type II cells are a target cell for HCoV-HKU1 infection in the lower respiratory tract, that type II alveolar cells are immune-competent in response to infection exhibiting a type III interferon and proinflammatory chemokine response, and that cell to cell spread may be a major factor for spread of infection. Furthermore, these studies demonstrate that human alveolar cells can be used to isolate and study novel human respiratory viruses that cause lower respiratory tract disease.

## Introduction

Respiratory tract cells are structurally and functionally different in the upper respiratory tract (nasal and sinusoidal epithelia), conducting airways (tracheal and bronchial epithelia), and alveoli (alveolar epithelia). Because they are the natural target cells for respiratory virus infection, primary human respiratory epithelial cell cultures provide the ideal *in vitro* systems for investigation of cell factors required for growth of respiratory human viruses, for analysis of their interactions with viruses and their innate immune responses to infection, and for isolation and propagation of novel respiratory pathogens. The alveolar epithelium consists of both alveolar type I cells (ATI), which make up 95% of the surface area of the lung, are terminally differentiated, non-dividing, and function in gas exchange and fluid homeostasis; and alveolar type II cells (ATII), which produce surfactant proteins and lipids, divide and differentiate to replace damaged ATI cells, participate in fluid homeostasis, and contribute to the innate defenses of the lung [1], [2], [3], [4]. Our laboratory has developed a system to isolate and culture primary ATII cells from human lungs and under specialized culture conditions transdifferentiate the ATII cells into an ATI-like phenotype [5].

In 2005 the fifth human coronavirus, HCoV-HKU1, was discovered by RT-PCR screening with conserved coronavirus primers on respiratory samples from adult patients with pneumonia that were negative for the severe acute respiratory syndrome coronavirus (SARS-CoV) [6]. To date, HCoV-HKU1 has been associated with both upper and lower respiratory tract illness in children and adults [7], [8], [9], [10], [11], [12]. Until recently, research on HCoV-HKU1 has been limited because it could not be isolated from clinical specimens in continuous cell lines *in vitro,* and there are no reports of HCoV-HKU1 infecting animals. Using primary human ciliated airway epithelial cell cultures, Pyrc et al isolated and propagated HCoV-HKU1, and more recently, Dijkman et al extended these observations to additional primary isolates of HCoV-HKU1 [13], [14]. Similarly, we developed a primary human bronchial-tracheal epithelial cell (HTBEC) culture system at the air-liquid interface (AL/I) and have successfully propagated HCoV-HKU1 from patient specimens. In parallel to these advances, and as an alternative approach to study HCoV-HKU1, we reasoned that HCoV-HKU1, like SARS-CoV [15], may be able to infect and be serially propagated in human alveolar cells since this virus is known to cause pneumonia in a subset of patients. Furthermore, we reasoned that infecting primary human alveolar cells would allow us to determine which subset of cells is susceptible to infection, and to characterize initial innate immune responses of alveolar epithelial cells to viruses that cause lower respiratory tract diseases. Here we demonstrate that HCoV-HKU1 can infect and be serially propagated in primary human alveolar type II cells but not in alveolar type I-like cells or alveolar macrophages at the air-liquid interface.

## Materials and Methods

### Isolation and Culture of Primary Cells from Human Lung

**Figure 1:**
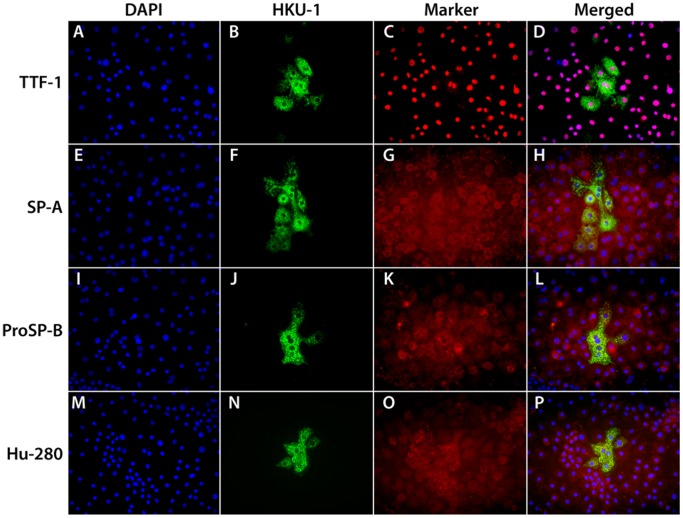
Immunofluorescent staining for HCoV-HKU1 spike protein and selected alveolar type II cell markers. The cells were grown under air/liquid conditions as described in the methods section, inoculated with HCoV-HKU1 and fixed 72 hours post inoculation. Panels A-D show staining for DAPI (A), HCoV-HKU1 (B), TTF-1 (C), and merged (D). Panels E-H show staining for pro DAPI (E), HCoV-HKU1 (F), SP-A (G), and merged (H). Panels I-L show staining for DAPI (I), HCoV-HKU1 (J), proSP-B (K), and merged (L). Panels M-P show staining for DAPI (M), HCoV-HKU1 (N), AT280 (Dobbs) (O), and merged (P). Cells that are infected with HCoV-HKU1 stain for the type II cell markers.

Human alveolar macrophages and alveolar cells were isolated as previously described [5], [16], [17]. Briefly, de-identified donor lungs that were not suitable for transplant were obtained through the National Disease Research Interchange (Philadelphia, PA) and the International Institute for the Advancement of Medicine (Edison, NJ). The isolation of cells was conducted as previously described with the exception that the type II cells were positively selected using EpCAM (CD326) magnetic beads (Miltenyi, Auburn, CA). Cells were plated in DMEM with 10% FBS on millicell inserts (Millipore, Bedford, MA) coated with a mixture of 80% rat tail collagen and 20% Matrigel (BD Biosciences, San Jose, CA). Cells were allowed to adhere to inserts for 2 days submerged in DMEM with 10% FBS, then cultured at an air-liquid interface in DMEM with 1% charcoal-stripped serum (CS-FBS) supplemented with keratinocyte growth factor (K) for 2 days, and then switched to media that additionally had isobutylmethylxanthine (I), 8-bromo-cAMP (A), and dexamethasone (D) for 2 days to achieve the ATII differentiated phenotype prior to infection (day 6 after plating). The alveolar macrophages and alveolar type I-like cells were cultured and characterized as reported previously [5], [16], [17]. Briefly, to transdifferentiate type II cells to type I–like cells, type II cells were plated on rat tail collagen–coated dishes at a density of 0.5–1.0×10^5^/cm^2^ in DMEM with 10% FBS (15). After 24–48 h the medium was changed to 5% FBS without additives. Type I-like cell phenotype was documented by positive staining for receptor for advanced glycation end products (RAGE, R & D systems, Minneapolis, MN) and epithelial membrane protein 2 (EMP2, Sigma-Aldrich, Inc, St.Louis, MO). For alveolar macrophages, the middle lobe of the lungs was perfused, and then lavaged with HEPES-buffered saline containing 2 mM EDTA and then HEPES-buffered saline alone. The macrophage purity of the adherent cells was nearly 100% and demonstrated by staining for CD 68 (DakoCytomation, Carpinteria,CA). The purity of the type II cells is demonstrated in Figure 1 and the level of surfactant protein expression has been published [5], [20], [25].

### Virus

Clinical nasopharyngeal specimens containing HCoV-HKU1 RNA were identified from pediatric patients with respiratory symptoms and submitted for testing to the clinical virology laboratory at the Children’s Hospital Colorado by xTag ® Respiratory Virus Panel, RVP (Luminex Molecular Diagnostics, Toronto CN). Aliquots of samples positive for HCoV-HKU1 RNA were stored in M4 viral transport media (Remel Laboratories, Lenexa, KS) at −70°C. Isolates of HCoV-HKU1 from five different patients (HKU1/DEN/2010/10, HKU1/DEN/2010/12, HKU1/DEN/2010/13, HKU1/DEN/2010/18, and HKU1/DEN/2010/21) were used for this study.

### Virus Infection and Propagation

Cells were inoculated with primary clinical isolates of HCoV-HKU1 or isolates of HCoV-HKU1 which had been passaged one time on primary, differentiated, human bronchial-tracheal epithelial cells (HTBEC) (P1 virus) (Lifeline Cell Technology, Walkersville, MD), at 1∶10 dilution in DMEM with 1% bovine serum albumin (BSA). After 4 hour incubation, the inoculum was removed and cells were incubated at 34^o^C. For cells kept at an air-liquid interface (AL/I), the apical surface of the cells were washed every 24 hours with DMEM +1% BSA and collected for PCR analysis. For cells kept immersed, the media was collected and replaced every 24 hours. Cell morphology was monitored daily to observe for any cytopathic effects. Three days after inoculation, cells were fixed with 100% methanol and viral antigens and cell markers were detected using immunostaining. HCoV-HKU1 was detected in infected cells using a rabbit polyclonal antibody or a mouse-monoclonal antibody directed against the HCoV-HKU1 viral spike glycoprotein developed in our laboratory and visualized using a goat anti-rabbit or goat anti-mouse antibody conjugated to ALEXA 488 fluorophore (Life Technologies, Grand Island, NY). Cells were also stained for cell type specific markers [ATII cells: SP-A (PE-10, a gift from Yoshio Kuroki, University of Sapporo, Japan), proSP-B (Millipore, Billerica, MA), TTF-1 (Leica Biosystems, Buffalo Grove, IL), and mouse IgM anti-Dobbs (Hu-280, a gift from Leland Dobbs, University of California – San Francisco); Basal cells: keratin-5 (Thermo Scientific, Pittsburgh, PA); Alveolar macrophages: CD68, (clone KP1, DAKO, Inc., Carpenteria, CA)]. Immunostained cells were imaged using a Zeiss Axioskop 2 fluorescent microscope with Axiovision software and a Zeiss LSM 700 laser scanning confocal microscope with Zen software (National Jewish Cytometry Core).

### Viral RNA Extraction and Real-time Quantitative Polymerase Chain Reaction (qPCR)

Viral RNA from pooled apical washes was extracted using Qiagen EZ1 Virus Mini Kits (Valencia, CA) on a BioRobot EZ1 Extractor (Qiagen, Valencia, CA) following the manufacturer’s instructions. Virus yield was determined by a quantitative real time RT-PCR (qRT-PCR) which targeted the HCoV-HKU1 polymerase 1b gene with comparison to copy number from a plasmid encoding the region recognized by this assay. The RT-PCRs were performed using a 1-step reverse-transcription PCR master mix (RNA UltraSense One-Step Quantitative RT-PCR System, Invitrogen Life Technologies, Carlsbad, CA). Reaction mixtures were prepared using a 500-nM final concentration of the forward primer (5′-TGGTGGCTGGGACGATATGT-3′), 250 nM of the reverse primer (5′-GGCATAGCACGATCACACTTAGG-3′), and 100 nM of the probe (5′-6FAM-ATAATCCCAACCCATRAG- minor groove binder nonfluorescent quencher-3′). 10 ul of RNA was added to 10 ul of master mix containing an additional 1.3 mM MgCl. RT-PCR was performed using the following cycling conditions: 50°C for 15 minutes, 95°C for 2 minutes, and 40 cycles of 95°C for 15 seconds, 60°C for 30 seconds [12].

### Cytokine Analysis

At 72 hours post-infection, cells were harvested for RNA analysis using the RNeasy RNA extraction kit (Qiagen, Valencia, CA), cDNA synthesis using qScript cDNA SuperMix (Quanta BioSciences, Gaithersburg, MD). To quantitate mRNAs encoding interferons, cytokines and chemokines, we used Taqman gene expression assays (Applied Biosystems, Life Technologies, Grand Island, NY) real-time PCR primers and probes [CXCL10 Hs00171042_m1, RANTES (CCL5) Hs00982282_m1, CYB Hs00168719_m1], or primers and probes synthesized in house: IFN-β (forward primer: 5′-CTTACAGGTTACCTCCGAAACTGAA-3′, reverse primer: 5′-TTGAAGAATGCTTGAAGCAATTGT-3′, probe: ATCTCCTAGCCTGTGCCTCTGGGACTGT), IL-29 (forward primer: 5′-GGGAACCTGTGTCTGAGAACGT-3′, reverse primer: 5′-GAGTAGGGCTCAGCGCATAAATA-3′, probe: 5′-CTGAGTCCACCTGACACCCCACACCT-3′), and IL-6 (forward primer: 5′-CCAGGAGCCCAGCTATGAAC-3′, and reverse primer: 5′- CCCAGGGAGAAGGCAACTG-3′, probe: 5′- CCTTCTCCACAAGCGCCTTCGGTT-3′). 15 uL reaction mixtures were prepared using 2 uL of cDNA, with a final probe concentration of 250 nM and primer concentration of 900 nM. Real-time PCR analysis was performed on Bio-Rad CFX96 instrument (Bio-Rad Laboratories, Hercules, CA) using the following cycling conditions: 50°C for 2 minutes, 95°C for 10 minutes, and 40 cycles of 95°C for 15 seconds and 60°C for 1 minute. Statistical analysis was performed using Prism software (GraphPad Software Inc., La Jolla, CA). Gene expression values were normalized to show expression relative to the cyclophilin B (CYB), a housekeeping gene.

### Ethics Statement

The Committee for the Protection of Human Subjects at National Jewish Health has deemed the use of human lung donor cells as nonhuman subject research, since there is no risk to donors and all donors are de-identified. Use of the banked virus specimens and clinical data for this research was approved by the Colorado Multiple Institutional Review Board.

## Results

### Clinical Characteristics of HCoV-HKU1 Viruses

The 5 clinical isolates of HCoV-HKU1 from Denver, CO, utilized in the study were part of a larger set of HCoV-HKU1 samples collected as part of a study on the epidemiology of CoV infections in pediatric patients detailed elsewhere [12], [18]. The clinical details of the patients from whom the isolates were obtained are summarized in Table 1. All of these HCoV-HKU1 isolates were HCoV-HKU1 genotype A.

**Table 1:** Clinical and demographic characteristics of patients from Colorado HCoV-HKU1 isolates that were successfully propagated in primary human alveolar type II cells.

Sample	SampleDate	Age	Sex	Underlying Medical Condition	Clinical Presentation	DischargeDiagnosis	HCoV-HKU1 genotype
HKU1/DEN/2010/10	1/6/2010	21 yr	F	heart transplant	fever, chest pain, cough, tachypnea	DVT+viral URTI	A
HKU1/DEN/2010/12	1/9/2010	unk	unk	none	unk	unk	A
HKU1/DEN/2010/13	1/8/2010	2 yo	M	CHD	increased O_2_ requirement	hypoxemia	A
HKU1/DEN/2010/18	1/22/2010	2 mo	F	none	fever, rhinorrhea	viral URTI	A
HKU1/DEN/2010/21	1/19/2010	16 yo	M	ALL	unk	bronchitis	A

ALL = acute lymphoblastic leukemia; CHD = congenital heart disease; DVT = deep venous thrombus; unk = unknown; URTI = upper respiratory tract infection.

### HCoV-HKU1 Infection of Primary Human Alveolar Cells

**Figure 2:**
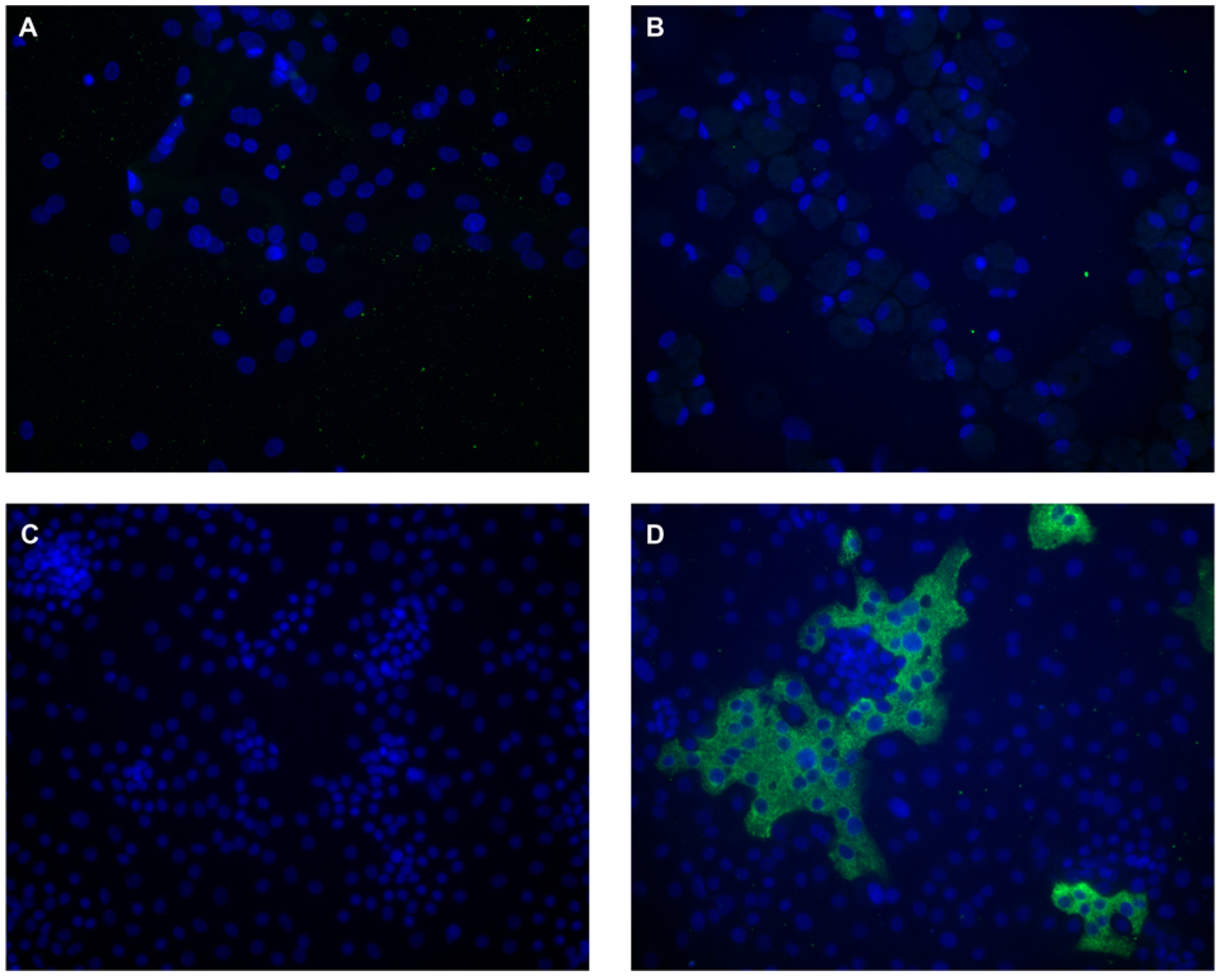
HCoV-HKU1 Infection of Primary Human Alveolar Cells. Cells were inoculated with media alone or a 1∶10 dilution of the clinical isolate HKU1/DEN/2010/21 at 34^o^C, maintained at the air-liquid interface, and fixed 96 hours post infection. Cell cultures were immunolabeled with polyclonal rabbit antibodies to purified HCoV-HKU1 spike protein and fluorescein labeled anti rabbit IgG (green). Nuclei were stained with DAPI (blue). Panels A and B shows infection of type I-like alveolar cells and alveolar macrophages, respectively. Panels C-D show mock and HCoV-HKU1 infection of alveolar type II cells, respectively. Neither the type 1-like cells nor alveolar macrophages were susceptible to infection. In contrast, alveolar type II cells supported infection with HCoV-HKU1.

To determine which subset of primary human alveolar cells are susceptible to HCoV-HKU1 infection, human alveolar type I-like cells, alveolar type II cells, and alveolar macrophages were tested. In addition, two different conditions for cell growth were compared, submerged cultures versus cultures at the air-liquid interface (AL/I). Cultures were inoculated with primary clinical isolates of HCoV-HKU1 or passage 1 (P1) virus. Titers of viral RNA in the wash from the apical surface of the cells were determined by qRT-PCR at the indicated time points, and cultures were fixed and immunolabeled with antibodies to the HCoV-HKU1 spike glycoprotein to identify infected cells. Neither the submerged nor air-liquid interface type 1-like cells nor alveolar macrophages were susceptible to infection by HCoV-HKU1 (Figure 2). In the submerged culture of type II cells only a few cells were infected with HCoV-HKU1. In contrast, type II cells maintained at the air-liquid interface supported infection, although the level of infection varied with individual subjects.

**Figure 3:**
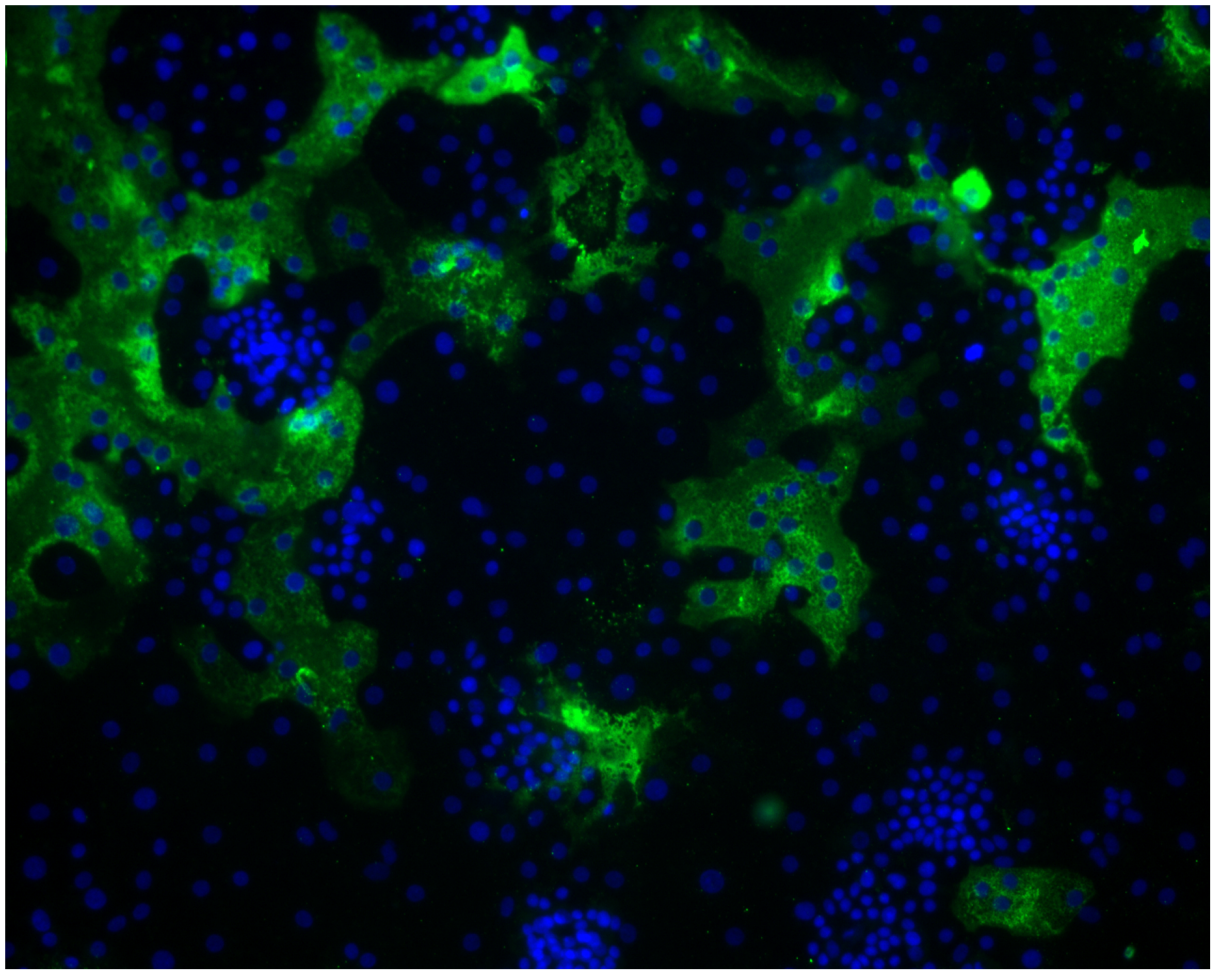
Formation of large syncytia of primary human alveolar type II cells infected with HCoV-HKU1. Cells were inoculated with a 1∶10 dilution of the clinical isolate HKU1/DEN/2010/21 at 34^o^C and fixed 120 hours post infection. Type II cell cultures were immunolabeled with polyclonal rabbit antibodies to purified HCoV-HKU1 spike protein and fluorescein labeled anti rabbit IgG (green). Nuclei were stained with DAPI (blue). Viral antigen is seen only within the cytoplasm of the cells. Efficient infection with cell to cell spread and formation of large, multinucleated giant cells is clearly evident.

Type II cells infected with HCoV-HKU1 showed formation of large syncytia indicating cell to cell spread may be a major factor for virus spread within the lung as it is for respiratory syncytial virus and parainfluenza viruses (Figures 2 and 3). This pattern of susceptibility of ATII cells at the AL/I to HCoV-HKU1 infection was reproducible in most human donors, since cultures from 17 of 20 different donors tested were susceptible to infection with HCoV-HKU1, although cells from different donors demonstrated significant variation in the percent of HCoV-HKU1 infected cells. Similarly, all 5 of our clinical HCoV-HKU1 specimens were able to infect type II cells at the air-liquid interface.

The ATII cells had a morphological appearance of a cobblestone epithelial monolayer, typical for type II cells. These cells were fluorescently labeled with antibodies to surfactant protein A (SP-A), pro-surfactant protein B (proSP-B), thyroid transcription factor–1 (TTF-1), and a type II specific transmembrane protein (Hu-280) using an antibody obtained from Dr. Leland Dobbs, University of California, San Francisco [19] (Figure 1). There were a few clumps of airway basal cells in these cultures. These cells stained for keratin 5 but were not infected by HCoV-HKU1. Other than the formation of large syncytia, cytopathic effects (CPE) were not detectable in any of the type II cell culture at the AL/I.

### Kinetics of HCoV-HKU1 Replication in Type II Human Alveolar Cells at AL/I

**Figure 4:**
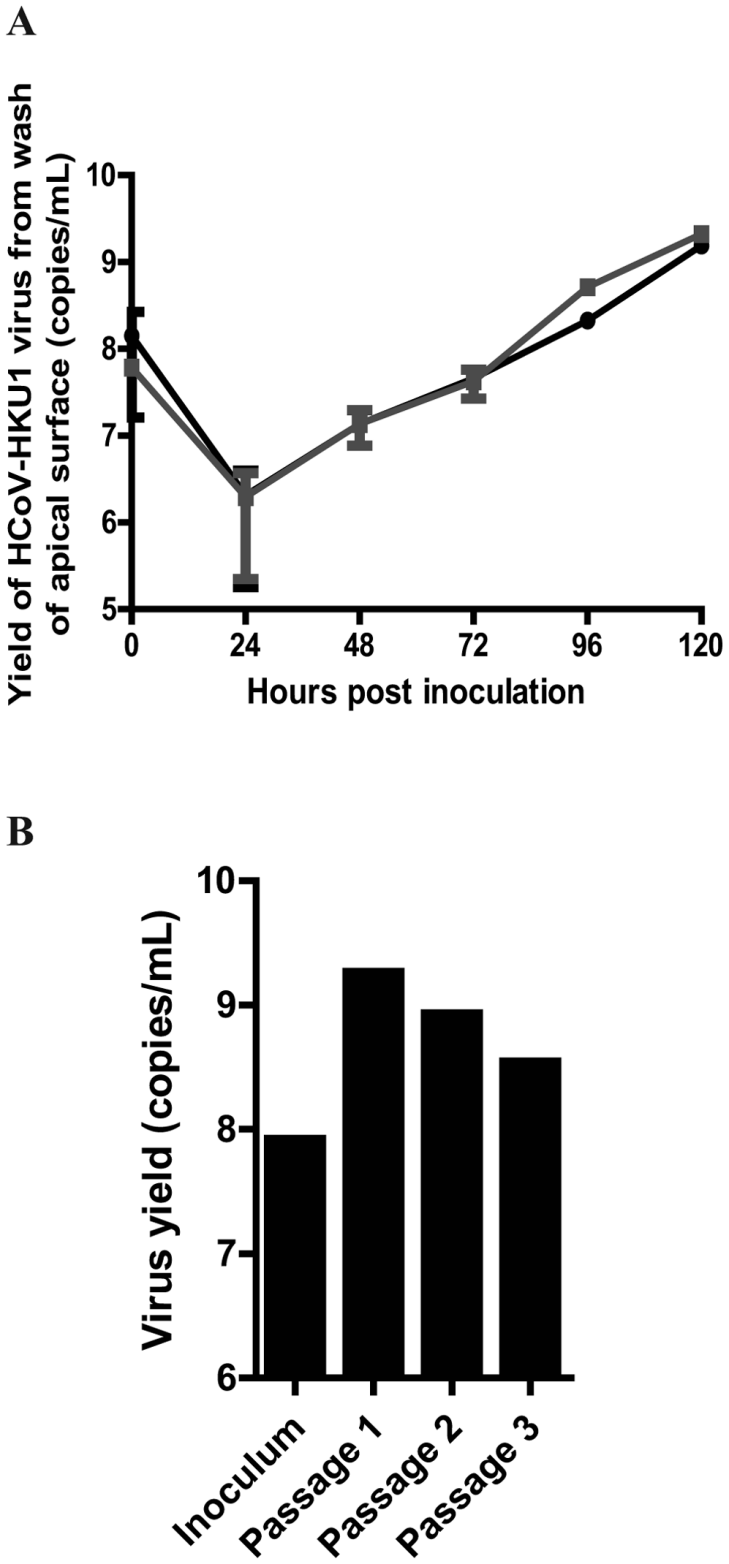
(A) Replication kinetics of HCoV-HKU1 in human alveolar type II cells. Cultures were inoculated with a primary clinical isolate of HCoV-HKU1. Data represent RT-PCR of apical washes from HCoV-HKU1 infected cells harvested at the indicated time points postinoculation. Data lines represent two independent experiments performed in duplicate (mean±standard deviation). (**B**) Propagation of an HKU1 clinical isolate in human alveolar type II cells at the air liquid interface. Alveolar cells were inoculated with diluted nasal pharyngeal washes for the first passage and with apical washed 120 hr postinoculation for subsequent passages. The bars represent real-time RT-PCR analysis of apical washes at 120 hr. IFA performed at 120 hours confirmed the presence of infected cells at each passage.

To examine the kinetics of HCoV-HKU1 replication in type II alveolar cells, primary clinical specimens containing HCoV-HKU1 RNA were used to inoculate the apical surface of type II alveolar cells for 4 hrs and then returned to the AL/I again. Apical surfaces were washed 24, 48, 72, 96, and 120 hrs post inoculation and assayed for HCoV-HKU1 RNA by qRT-PCR. Viral genome copies increased approximately 1000 fold during the course of the infection indicating productive infection and replication (Figure 4a).

To determine if human alveolar cells could be utilized to propagate HCoV-HKU1, apical surfaces were inoculated and incubated for 4 hr at 34^o^C with a clinical sample of HCoV-HKU1 and then removed. The apical surfaces were then washed every 24 hr for the next 5 days. Serial passage of HCoV-HKU1 infection was performed by inoculating naïve cultures with 100 µL of the day 5 (120 hr post inoculation) apical wash from the previous passage. Viral genome copy numbers were maintained over the course of three passages (Figure 4b). Immunofluorescence assays (IFA) with antibodies to the HCoV-HKU1 spike glycoprotein performed at 120 hours confirmed the presence of infected cells at the end of each passage. These experiments demonstrated that genome copies detected in the apical wash were infectious and did not represent defective particles, confirming that HCoV-HKU1 was truly replicating in type II alveolar cells at the AL/I.

### Innate Immune Response Elicited by HCoV-HKU1 Infection in Type II Human Alveolar Cells

**Figure 5:**
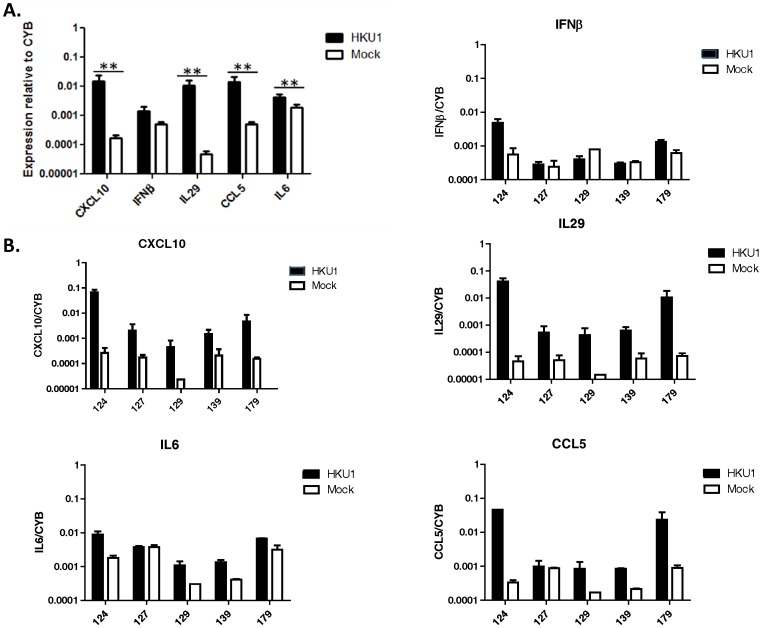
Analysis of anti-viral gene expression in human alveolar epithelial cells in response to HCoV-HKU1 infection. Total mRNA from HCoV-HKU1 and control cells was analyzed at 72 hours post inoculation for immune response genes: CXCL10, IFNβ, IL29 (IFNλ), CCL5 (RANTES) and IL6. Gene expression values were normalized to expression of the cyclophilin B (CYB), a housekeeping gene. Figure (A) shows the average results from 5 human donors (p-values from Wilcoxon signed rank test, **p≤0.05) and (B) shows the response to HCoV-HKU1 infection by individual donor. Error bars represent the standard deviation between donors (A) or replicates (B).

Because alveolar type II cells at the air-liquid interface were highly susceptible to HCoV-HKU1 infection, we could measure changes in the level of expression of select genes of the innate immune system in these cells in response to HCoV-HKU1 infection. At 72 hours post inoculation, HCoV-HKU1 infection induced markedly increased levels of mRNAs encoding type III interferons (IL29), interferon-responsive genes (CXCL10), proinflammatory chemokines (CCL5 and CXCL10), and IL-6. There was no significant increase in IFNβ, the only type I interferon reported to be expressed by alveolar type II cells [20] (Figure 5a). The levels of relative cellular gene expression varied significantly between human donors tested (Figure 5b). Thus, human alveolar type II cells induce significant innate, anti-viral responses to infection with HCoV-HKU1, but the extent of these responses varied in different humans with different levels of infection.

## Discussion

This is the first study to demonstrate infection of primary human alveolar type II cells at the AL/I with primary clinical specimens containing HCoV-HKU1 RNA. Human alveolar type II cells maintained at an air-liquid interface were highly susceptible to HCoV-HKU1 infection, but alveolar macrophages and type I-like cells were not susceptible to HCoV-HKU1. This supports our hypothesis that primary human alveolar cells can be a suitable culture system for isolation and propagation of novel human respiratory viruses that cause lower respiratory tract disease and which display a stringent degree of cell, tissue, and host specificity. Utilization of this culture system is appealing because it most closely represents the *in vivo* physiological conditions of the lung [2], [21]. The type II culture system allows identification of which terminal human respiratory epithelial cells are susceptible to infection and might provide insight into the spread of infections from the conducting airways required for the development of pneumonia and the acute respiratory distress syndrome (ARDS).

These results are agreement with what is known about the SARS-CoV, another beta-coronavirus (lineage b) that causes more severe lower respiratory tract disease than HCoV-HKU1. Autopsy studies of patients with SARS-CoV demonstrated expression of viral antigen in type II pneumocytes [22], [23], [24], and previous studies in our laboratory showed that *in vitro* cultures of human alveolar type II cells at the AL/I are susceptible to infection with SARS-CoV [15], [25]. Similarly, a recent study using human lung organ cultures demonstrated that various subtypes of human influenza A viruses, which varied in their pathogenic potential, all preferentially infected type II alveolar cells, with minimal infection of alveolar macrophages and no infection of alveolar type I cells [21]. Their study also highlighted that the different pathogenic potential of influenza A viruses was not attributed to differences in cellular tropism, as they all infected the same cells, but rather to the capacity of the viruses to productively replicate in type II alveolar cells. Overall, these studies emphasize the importance of ATII cells in the pathogenesis of viral induced lower respiratory tract disease. In contrast to these viruses, human coronavirus 229E, an alphacoronavirus, can readily infect human alveolar macrophages, but not type I-like or type II alveolar cells [17]. Interestingly, in contrast to SARS-CoV, HCoV-229E is a common cause of upper respiratory tract infection and rarely causes severe lower respiratory tract disease [26], [27]. The differences in clinical presentation between these coronavirus may be partially explained by whether that can infect type II alveolar cells.

Several observations regarding infection of the type II cells with HCoV-HKU1 are worth noting. Growing and infecting the cultures at an air-liquid interface, as opposed to keeping them submerged, greatly increased the susceptibility of type II cells to HCoV-HKU1. These results are identical to what we previously described for SARS-CoV [25]. Interestingly, there is very little fluid at the apical surface of the alveolar epithelium [28], [29], except in diseased states, and therefore the AL/I conditions more accurately approximate *in vivo* conditions for type II cells. Over the course of our experiments we infected cells from 20 different donors. Type II cells from 17 of these donors were susceptible to HCoV-HKU1 infection, although there was significant variability in the degree of susceptibility between donors. One possible explanation for these differences might be differential levels of receptor expression. As the receptor for HCoV-HKU1 is currently unknown we were not able to explore this hypothesis. Elucidating the mechanism(s) for the increased susceptibility in the AL/I conditions and the variability exhibited between donors might unravel important cellular determinants of infection.

Induction of syncytia formation by HCoV-HKU1 most likely indicates direct cell to cell spread of virus and undoubtedly plays an important role in the pathogenesis of HCoV-HKU1 in lung disease and escape from immune surveillance. Many infections with enveloped viruses can directly move from cell to cell by fusion of adjacent plasma membranes resulting in large, multinucleated giant cells. This ability confers multiple potential pathogenic advantages over cell free spread. Direct cell to cell spread is more efficient by eliminating the intrinsic barriers of fluid-phase diffusion required by cell free spread to encounter another target cells and engage the appropriate receptors. Movement of infection between cells also allows the virus to evade innate humoral and cellular defenses [30], [31]. Viral fusion with cell membranes of their target cells (virus-cell fusion) is an essential first step in infection for all coronaviruses. Syncytia formation is also commonly seen in other coronaviruses and plays an important step in viral pathogenesis [32], [33], [34]. Syncytia formation of dendritic cells resulting in cell death was recently shown to be important in an *in vitro* infection model of another human respiratory coronavirus, 229E [35]. Syncytia forming activity has been shown to greatly enhance the infectivity of avian coronavirus infectious bronchitis [36]. Modulation of syncytial formation helps to establish viral persistence in *in vitro* culture models of infection with the murine coronavirus, mouse hepatitis virus (MHV) [37]. Both virus-cell entry and syncytia formation are mediated by the coronavirus spike protein, a class 1 viral fusion protein that is a major determinant of cell and host specificity. These two important processes in the pathogenesis of HCoV-HKU1, however, may occur through different mechanisms as has been shown for SARS-CoV and MHV [38], [39], [40], [41].

The innate immune system serves as the first line of defense in protection of the host against viral infections and helps to shape the resultant adaptive immune response. The characteristics of the initial immune response can greatly impact the resulting clinical outcome [42]. Expression of pro-inflammatory cytokines in response to viral infection argues that type II alveolar cells are not only target cells for infection but are also immune-competent and may directly influence the immune response to viral infection. Many of the cytokine responses of the human type II alveolar cells at the AL/I to HCoV-HKU1 (induction of CXCL-10, IL29, and CCL5) were similar to those we previously described for influenza and SARS-CoV [20] [25]. One significant difference was a lack of expression of the type I interferon, IFNβ, which may reflect that our current assays were conducted at a later time point during the course of infection. Alternatively, it could be due to the presence of unique viral proteins which can suppress the IFN response. Another difference was that the degree of induction of these cytokines was overall approximately 10 fold less for HCoV-HKU1 compared with influenza and SARS-CoV. This may have important implications as recently it was shown that the level of cytokine responses in type II alveolar cells induced by different strains of influenza A viruses were correlated with degree of pathogenicity [21]. Similarly, the differences in cytokine responses exhibited by different human donors might partially explain the variability in clinical outcomes to HCoV-HKU1 infections.

In summary, HCoV-HKU1 can infect, be serially propagated, and induce an anti-viral response in human alveolar type II cells maintained at an air-liquid interface. Type II cells infected with HCoV-HKU1 demonstrated syncytia formation indicating cell to cell spread may be a major factor for virus spreading *in vivo*, suggesting a potential mechanism for evasion of host immune surveillance. These experiments demonstrate that HCoV-HKU1 has strong tropism for type II alveolar cells and demonstrate the ability to use human alveolar cells to isolate and study novel human respiratory viruses that cause lower respiratory tract disease which display a stringent degree of cell, tissue, and host specificity.

## References

[1] Miura TA, Wang J, Mason RJ, Holmes KV (2006) Rat coronavirus infection of primary rat alveolar epithelial cells. Adv Exp Med Biol 581: 351–356. 17037558 10.1007/978-0-387-33012-9_62 PMC7123676

[2] Miura TA, Holmes KV (2009) Host-pathogen interactions during coronavirus infection of primary alveolar epithelial cells. J Leukoc Biol 86: 1145–1151. 19638499 10.1189/jlb.0209078 PMC2774885

[3] Mason RJ (2006) Biology of alveolar type II cells. Respirology 11 Suppl: S12–15 10.1111/j.1440-1843.2006.00800.x 16423262

[4] Williams MC (2003) Alveolar type I cells: molecular phenotype and development. Annu Rev Physiol 65: 669–695. 12428023 10.1146/annurev.physiol.65.092101.142446

[5] Wang J, Edeen K, Manzer R, Chang Y, Wang S, et al (2007) Differentiated human alveolar epithelial cells and reversibility of their phenotype in vitro. Am J Respir Cell Mol Biol 36: 661–668. 17255555 10.1165/rcmb.2006-0410OC PMC1899340

[6] Woo PC, Lau SK, Tsoi HW, Huang Y, Poon RW, et al (2005) Clinical and molecular epidemiological features of coronavirus HKU1-associated community-acquired pneumonia. J Infect Dis 192: 1898–1907. 16267760 10.1086/497151 PMC7110183

[7] Woo PC, Lau SK, Chu CM, Chan KH, Tsoi HW, et al (2005) Characterization and complete genome sequence of a novel coronavirus, coronavirus HKU1, from patients with pneumonia. J Virol 79: 884–895. 15613317 10.1128/JVI.79.2.884-895.2005 PMC538593

[8] Vabret A, Dina J, Gouarin S, Petitjean J, Corbet S, et al (2006) Detection of the new human coronavirus HKU1: a report of 6 cases. Clin Infect Dis 42: 634–639. 16447108 10.1086/500136 PMC7107802

[9] Sloots TP, McErlean P, Speicher DJ, Arden KE, Nissen MD, et al (2006) Evidence of human coronavirus HKU1 and human bocavirus in Australian children. J Clin Virol 35: 99–102. 16257260 10.1016/j.jcv.2005.09.008 PMC7108338

[10] Kuypers J, Martin ET, Heugel J, Wright N, Morrow R, et al (2007) Clinical disease in children associated with newly described coronavirus subtypes. Pediatrics 119: e70–76. 17130280 10.1542/peds.2006-1406

[11] Esper F, Weibel C, Ferguson D, Landry ML, Kahn JS (2006) Coronavirus HKU1 infection in the United States. Emerg Infect Dis 12: 775–779. 16704837 10.3201/eid1205.051316 PMC3374449

[12] Dominguez SR, Robinson CC, Holmes KV (2009) Detection of four human coronaviruses in respiratory infections in children: a one-year study in Colorado. J Med Virol 81: 1597–1604. 19626607 10.1002/jmv.21541 PMC2879166

[13] Dijkman R, Jebbink MF, Koekkoek SM, Deijs M, Jonsdottir HR, et al.. (2013) Isolation and characterization of current human coronavirus strains in primary human epithelia cultures reveals differences in target cell tropism. J Virol. 10.1128/JVI.03368-12 PMC3648119 23427150

[14] Pyrc K, Sims AC, Dijkman R, Jebbink M, Long C, et al (2010) Culturing the unculturable: human coronavirus HKU1 infects, replicates, and produces progeny virions in human ciliated airway epithelial cell cultures. J Virol 84: 11255–11263. 20719951 10.1128/JVI.00947-10 PMC2953148

[15] Mossel EC, Wang J, Jeffers S, Edeen KE, Wang S, et al (2008) SARS-CoV replicates in primary human alveolar type II cell cultures but not in type I-like cells. Virology 372: 127–135. 18022664 10.1016/j.virol.2007.09.045 PMC2312501

[16] Wang J, Oberley-Deegan R, Wang S, Nikrad M, Funk CJ, et al (2009) Differentiated human alveolar type II cells secrete antiviral IL-29 (IFN-lambda 1) in response to influenza A infection. J Immunol 182: 1296–1304. 19155475 10.4049/jimmunol.182.3.1296 PMC4041086

[17] Funk CJ, Wang J, Ito Y, Travanty EA, Voelker DR, et al (2012) Infection of human alveolar macrophages by human coronavirus strain 229E. J Gen Virol 93: 494–503. 22090214 10.1099/vir.0.038414-0 PMC3352353

[18] Dominguez SR, Sims GE, Wentworth DE, Halpin RA, Robinson CC, et al.. (2012) Genomic Analysis of 16 Colorado Human NL63 Coronaviruses Identifies a New Genotype, High Sequence Diversity in the N-terminal Domain of the Spike Gene, and Evidence of Recombination. J Gen Virol. 10.1099/vir.0.044628-0 PMC4091283 22837419

[19] Fang X, Song Y, Hirsch J, Galietta LJ, Pedemonte N, et al (2006) Contribution of CFTR to apical-basolateral fluid transport in cultured human alveolar epithelial type II cells. Am J Physiol Lung Cell Mol Physiol 290: L242–249. 16143588 10.1152/ajplung.00178.2005

[20] Wang J, Nikrad MP, Phang T, Gao B, Alford T, et al (2011) Innate immune response to influenza A virus in differentiated human alveolar type II cells. Am J Respir Cell Mol Biol 45: 582–591. 21239608 10.1165/rcmb.2010-0108OC PMC3175576

[21] Weinheimer VK, Becher A, Tonnies M, Holland G, Knepper J, et al (2012) Influenza A Viruses Target Type II Pneumocytes in the Human Lung. J Infect Dis 206: 1685–1694. 22829640 10.1093/infdis/jis455 PMC7107318

[22] Chow KC, Hsiao CH, Lin TY, Chen CL, Chiou SH (2004) Detection of severe acute respiratory syndrome-associated coronavirus in pneumocytes of the lung. Am J Clin Pathol 121: 574–580. 15080310 10.1309/C0EDU0RAQBTXBHCE PMC7109992

[23] Shieh WJ, Hsiao CH, Paddock CD, Guarner J, Goldsmith CS, et al (2005) Immunohistochemical, in situ hybridization, and ultrastructural localization of SARS-associated coronavirus in lung of a fatal case of severe acute respiratory syndrome in Taiwan. Hum Pathol 36: 303–309. 15791576 10.1016/j.humpath.2004.11.006 PMC7112064

[24] Ye J, Zhang B, Xu J, Chang Q, McNutt MA, et al (2007) Molecular pathology in the lungs of severe acute respiratory syndrome patients. Am J Pathol 170: 538–545. 17255322 10.2353/ajpath.2007.060469 PMC1851867

[25] Qian Z, Travanty EA, Oko L, Edeen K, Berglund A, et al.. (2013) Innate Immune Response of Human Alveolar Type II Cells Infected with SARS-Coronavirus. American Journal of Respiratory and Critical Care Medicine. 10.1165/rcmb.2012-0339OC PMC3727876 23418343

[26] Heikkinen T, Jarvinen A (2003) The common cold. Lancet 361: 51–59. 12517470 10.1016/S0140-6736(03)12162-9 PMC7112468

[27] Holmes KV (2001) Coronaviruses. In: D. Knipe ea, editor. Fields' Virology. 4th ed. Philadelphia: Lippincott Williams and Wilkins. 1187–1203.

[28] Fehrenbach H (2001) Alveolar epithelial type II cell: defender of the alveolus revisited. Respir Res 2: 33–46. 11686863 10.1186/rr36 PMC59567

[29] Mason RJ, Williams MC, Widdicombe JH, Sanders MJ, Misfeldt DS, et al (1982) Transepithelial transport by pulmonary alveolar type II cells in primary culture. Proc Natl Acad Sci U S A 79: 6033–6037. 6964398 10.1073/pnas.79.19.6033 PMC347046

[30] Sattentau Q (2008) Avoiding the void: cell-to-cell spread of human viruses. Nat Rev Microbiol 6: 815–826. 18923409 10.1038/nrmicro1972

[31] Sattentau QJ (2011) The direct passage of animal viruses between cells. Curr Opin Virol 1: 396–402. 22440841 10.1016/j.coviro.2011.09.004

[32] Frana MF, Behnke JN, Sturman LS, Holmes KV (1985) Proteolytic cleavage of the E2 glycoprotein of murine coronavirus: host-dependent differences in proteolytic cleavage and cell fusion. J Virol 56: 912–920. 2999444 10.1128/jvi.56.3.912-920.1985 PMC252664

[33] Yamada Y, Liu DX (2009) Proteolytic activation of the spike protein at a novel RRRR/S motif is implicated in furin-dependent entry, syncytium formation, and infectivity of coronavirus infectious bronchitis virus in cultured cells. J Virol 83: 8744–8758. 19553314 10.1128/JVI.00613-09 PMC2738192

[34] Nakagaki K, Taguchi F (2005) Receptor-independent spread of a highly neurotropic murine coronavirus JHMV strain from initially infected microglial cells in mixed neural cultures. J Virol 79: 6102–6110. 15857995 10.1128/JVI.79.10.6102-6110.2005 PMC1091713

[35] Mesel-Lemoine M, Millet J, Vidalain PO, Law H, Vabret A, et al (2012) A human coronavirus responsible for the common cold massively kills dendritic cells but not monocytes. J Virol 86: 7577–7587. 22553325 10.1128/JVI.00269-12 PMC3416289

[36] Yamada Y, Liu XB, Fang SG, Tay FP, Liu DX (2009) Acquisition of cell-cell fusion activity by amino acid substitutions in spike protein determines the infectivity of a coronavirus in cultured cells. PLoS One 4: e6130. 19572016 10.1371/journal.pone.0006130 PMC2700284

[37] Mizzen L, Cheley S, Rao M, Wolf R, Anderson R (1983) Fusion resistance and decreased infectability as major host cell determinants of coronavirus persistence. Virology 128: 407–417. 6310865 10.1016/0042-6822(83)90266-0 PMC7130467

[38] Simmons G, Bertram S, Glowacka I, Steffen I, Chaipan C, et al (2011) Different host cell proteases activate the SARS-coronavirus spike-protein for cell-cell and virus-cell fusion. Virology 413: 265–274. 21435673 10.1016/j.virol.2011.02.020 PMC3086175

[39] Heald-Sargent T, Gallagher T (2012) Ready, set, fuse! The coronavirus spike protein and acquisition of fusion competence. Viruses 4: 557–580. 22590686 10.3390/v4040557 PMC3347323

[40] Follis KE, York J, Nunberg JH (2006) Furin cleavage of the SARS coronavirus spike glycoprotein enhances cell-cell fusion but does not affect virion entry. Virology 350: 358–369. 16519916 10.1016/j.virol.2006.02.003 PMC7111780

[41] de Haan CA, Stadler K, Godeke GJ, Bosch BJ, Rottier PJ (2004) Cleavage inhibition of the murine coronavirus spike protein by a furin-like enzyme affects cell-cell but not virus-cell fusion. J Virol 78: 6048–6054. 15141003 10.1128/JVI.78.11.6048-6054.2004 PMC415802

[42] Rouse BT, Sehrawat S (2010) Immunity and immunopathology to viruses: what decides the outcome? Nat Rev Immunol 10: 514–526. 20577268 10.1038/nri2802 PMC3899649

